# C1q and Mannose-Binding Lectin Interact with CR1 in the Same Region on CCP24-25 Modules

**DOI:** 10.3389/fimmu.2018.00453

**Published:** 2018-03-07

**Authors:** Mickaël Jacquet, Gianluca Cioci, Guillaume Fouet, Isabelle Bally, Nicole M. Thielens, Christine Gaboriaud, Véronique Rossi

**Affiliations:** ^1^Univ. Grenoble Alpes, CEA, CNRS, IBS, Grenoble, France

**Keywords:** complement, C1q, CR1/CD35, CCP modules, protein engineering, interaction, surface plasmon resonance, receptor

## Abstract

Complement receptor type 1 (CR1) is a multi modular membrane receptor composed of 30 homologous complement control protein modules (CCP) organized in four different functional regions called long homologous repeats (LHR A, B, C, and D). CR1 is a receptor for complement-opsonins C3b and C4b and specifically interacts through pairs of CCP modules located in LHR A, B, and C. Defense collagens such as mannose-binding lectin (MBL), ficolin-2, and C1q also act as opsonins and are involved in immune clearance through binding to the LHR-D region of CR1. Our previous results using deletion variants of CR1 mapped the interaction site for MBL and ficolin-2 on CCP24-25. The present work aimed at deciphering the interaction of C1q with CR1 using new CR1 variants concentrated around CCP24-25. CR1 bimodular fragment CCP24-25 and CR1 CCP22-30 deleted from CCP24-25 produced in eukaryotic cells enabled to highlight that the interaction site for both MBL and C1q is located on the same pair of modules CCP24-25. C1q binding to CR1 shares with MBL a main common interaction site on the collagen stalks but also subsidiary sites most probably located on C1q globular heads, contrarily to MBL.

## Introduction

C1q is a defense collagen that plays a crucial role in innate immunity. Beside its implication in the initiation of the classical pathway of complement, in association with its cognate C1r and C1s serine proteases, it is involved in a number of other non-complement functions, such as immune complex clearance, phagocytosis, cytokine regulation, and immune effector mechanisms mediation (1–4).

C1q is a complex molecule of 460 kDa, resembling a bunch of flowers made from the association of three different polypeptide chains, A, B, and C, organized into six heterotrimeric subunits. The overall molecule presents two distinct regions, with six globular heads (GR) on one end (flowers) and six collagenic stalks (CLF) assembled into a bundle of fibers, on the other end (5, 6). Because of its multimeric organization, C1q acts as a multipattern recognition molecule, as well as other members of the defense collagens familly, mannose-binding lectin (MBL), and ficolins (7, 8). Their anchorage on some of their targets is made possible by this multimeric scaffold that can provide avidity and versatile interactions with the globular heads and/or the collagen stalks. Therefore, the diversity of ligands for C1q relates in part to its GR and C1q tail domains, which bind specific cell surface receptors to regulate innate immunity. Most receptors described for C1q GR and CLF are involved in the phagocytic uptake by macrophages of self and non self-products. They include LDL receptor-related protein 1 (LRP1/CD91), calreticulin (CRT), gC1qR, receptor for advanced glycation end products (RAGE), leukocyte associated immunoglobulin-like receptor 1 (LAIR-1/CD305), sialic-acid-binding immunoglobulin-like lectin-3 (Siglec-3/CD33), and complement receptor 1 (CR1/CD35) (9–14). Some of these receptors interact with C1q GRs (RAGE, CD33, gC1qR) or with C1q CLF (LAIR-1), or with both C1q regions (CRT). Nevertheless, most of these interactions are not completely characterized yet and might differ according to the physiological context. In the case of CR1, it has been proposed that the interaction is mediated by C1q CLF (15, 16).

Complement receptor 1 (CR1/CD35) is a large multimodular type 1 transmembrane glycoprotein exposed at the surface of a wide range of cells. It is predominantly expressed on erythrocytes and most peripheral blood leukocytes and is mainly involved in the clearance from the blood stream of complement opsonized components (17–20). CR1 has also a major role in controling the complement-mediated attack. It is implicated in the acceleration of complement convertases decay and cooperates as a cofactor of factor I in the cleavage of C3b and C4b. Its crucial role in the protection of renal epithelial cells by reducing the level of complement deposition triggered by the classical and lectin pathways has been reported recently (21).

The extracellular region of the most common polymorphic size variant of CR1 is composed of 30 independent folding units called complement control protein repeats (CCP) organized in four long homologous repeats (LHR) A, B, C, and D. CR1 binds opsonins C3b and C4b specifically through pairs or triplets of consecutive CCP modules, CCP8-9 and CCP15-17 in LHR-B and C (C3b) and CCP1-2 in LHR A (C4b) (22–25). CR1 also binds C1q, MBL, and ficolin-2, more recently shown to also act as CR1-interacting opsonins specific of the LHR-D region (15, 26). The binding of MBL to CR1 involves its collagen-like regions at a site that is located at, or close to the site that interacts with its associated serine proteases (26). The opsonic function of MBL might, therefore, implicate a protease-free molecule linked to a target through its recognition domains and to cell surface CR1 through its collagen stalks.

Results from our laboratory indicated using a range of size variants of CR1 LHR-D that MBL and ficolin-2 bind to CCP24-25 (26). In the present study, we extend our previous findings by investigating more precisely the site of interaction for MBL using purified CCP24-25 and we highlight that C1q interacts with the same pair of modules. Moreover, we provide evidence that C1q binds CR1 through its collagen stalks but also through subsidiary sites, most probably located in its globular regions.

## Materials and Methods

### Proteins and Reagents

C1q was purified from human serum and quantified as previously described (27). Human serum was obtained from the Etablissement Français du Sang (EFS) Rhône-Alpes (agreement number 14-1940 regarding its use in research). C1q collagen stalks (CLF) and globular heads (GR) were prepared according to Tacnet-Delorme et al. (28). Recombinant MBL was provided by Natimmune (Copenhagen, Denmark) and quantified as previously described (29). Recombinant MBL-associated serine protease (MASP)-3 was obtained according to the procedure of Jacquet et al. (26). Recombinant soluble human CR1 (sCR1) was purchased from R&D Systems Europe. Oligonucleotides were from Eurogentec. Restriction and modification enzymes were from New England Biolabs. All CR1 variants constructs were engineered using site-directed mutagenesis with the QuickChange II XL kit (Agilent Technologies). For protein quantification, Mw and A_1%,1 cm_ were, respectively, for C1q (459,300; 6.8), CLF (189,900; 2.1), GR (48,000; 9.3), dimeric MASP-3 (175,200; 13.5), and tetrameric MBL (305,400; 7.8).

### Production of the CR1 CCP22-30 His_6_ Variants in Insect Cells

The CR1 CCP22-30 His_6_ fragment, its deletion variants ΔCCP22-23, ΔCCP26-30, ΔCCP25-26, and fragments CCP25-26 and CCP24-25 were produced in *Trichoplusia ni* insect cells, purified, characterized, and quantified as described in Ref. (26). The CCP24-25 His_6_ plasmid was generated by site-directed mutagenesis (Quickchange XLII, Agilent) from the template pNT-Bac-CR1 CCP22-30 His_6_, using an optimized procedure for large insertion/deletion (30). Purification of CCP24-25 on a Ni NTA column (His-select, Sigma Aldrich), was achieved as described previously (26). The concentration of the purified CR1 CCP24-25 variant was estimated using the absorption coefficient A_1%,1 cm_ at 280 nm of 10.5 calculated using the PROTPARAM program on the Expasy Server^1^, and an experimental molecular weight determined by MALDI mass spectrometry of 18,500 Da.

### Production of CR1 CCP22-30 His_6_ and CR1 CCP22-30 ΔCCP24-25 in Mammalian Cells

A 6 × His-Tag was inserted at the C-terminal end of CR1 CCP22-30 by site-directed mutagenesis using the pcDNA3.1 CR1 CCP22-30 construct as a template and the protocol described for CCP24-25 engineering. Using the same mutagenesis protocol, the ΔCCP24-25 deletion construct was then obtained by deletion of the CCP24-25 coding sequence from the pcDNA3.1 CR1 CCP22-30 His_6_ template. Purification of the secreted CR1 fragments transiently produced in Freestyle 293-F cells (4 days) was achieved as described previously (26). The concentration of purified CR1 CCP22-30 and CR1 ΔCCP24-25 was determined using respective Mw obtained by MALDI mass spectrometry of 78,752 ± 78 and 57,550 ± 57 Da and calculated A_1%, 1 cm_ at 280 nm of, respectively, 13.5 and 14.1.

### Surface Plasmon Resonance (SPR) Analyses and Data Evaluation

Multiple cycle interaction analyses and competition experiments were performed on a BIAcore 3000 instrument (GE Healthcare). Recombinant soluble CR1 (sCR1), CR1 CCP22–30, and its size variants were covalently immobilized on CM5 sensor chips in 10 mM HEPES, 150 mM NaCl, 0.005% surfactant P20, pH 7.4 (HBS-P) using the amine coupling chemistry according to the manufacturer’s instructions (GE Healthcare). The protein ligands were diluted in 10 mM sodium acetate, pH 4.2 at 25 µg/ml (sCR1), 20 µg/ml (CR1 CCP22-30), and 5 µg/ml (deletion variants and bimodular CCP fragments). Binding was measured at a flow rate of 20 µl/min in HBS-P containing 3 mM EDTA for MBL and in 50 mM triethanolamine-HCl (TEA), 150 mM NaCl, 1 mM CaCl_2_, 0.005% surfactant P20, pH 7.4 for C1q. Sixty microliters of each soluble analyte at desired concentrations were injected over surfaces with immobilized sCR1 [9,500 resonance units (RU)], CR1 CCP22–30 or CR1 ΔCCP24-25 (1,500 to 4,500 RU) and CCP24-25 (1,000 RU). A flow cell submitted to the coupling steps without immobilized protein was used as blank, and the specific binding signal was obtained by subtracting the background signal over the blank surface. For competition assays, C1q was pre incubated for 20 min at room temperature with recombinant MASP-3 in 50 mM TEA, 150 mM NaCl, 1 mM CaCl_2_ pH 7.4 before injection. Regeneration of the surfaces was achieved by 10 µl injections of 1 M NaCl, 10 mM EDTA. Kinetic data were analyzed by global fitting to a 1:1 Langmuir binding model of both the association and dissociation phases for at least five analyte concentrations simultaneously, using the BIAevaluation 3.2 software (GE Healthcare). Buffer blanks were subtracted from the data sets used for kinetic analysis (double referencing). The apparent equilibrium dissociation constants (*K*_D_) were calculated from the ratio of the dissociation and association rate constants (*k*_d_/*k*_a_). Chi^2^ values were below 7 in all cases.

Single cycle interaction analyses were performed on a T200 instrument (GE Healthcare). CR1 CCP22-30 and its deletion fragment ΔCCP24-25 were diluted to 20 µg/ml in 10 mM sodium acetate, pH 4.2, and immobilized on a CM5 (series S) sensor chip (GE Healthcare) in HBS-P using the amine coupling chemistry according to the manufacturer’s instructions (GE Healthcare). The reference surface was prepared using the same procedure except that the protein solution was replaced by buffer. The immobilization levels for CR1 CCP22-30 and ΔCCP24-25 were, respectively, 3,000 and 2,200 RU. C1q and MBL binding was measured in 50 mM TEA, 150 mM NaCl, 0.05% surfactant P20 pH 7.4 containing 1 mM CaCl_2_ for C1q or 3 mM EDTA for MBL at a flow rate of 20 µl/min. The signals recorded on the reference flow cell were subtracted from those obtained on immobilized CR1 fragments.

### Interaction Studies Using Solid-Phase Binding Assays

96-well microtiter plates (Greiner Bio-One) were coated with 3.4 pmol of each CR1 variant in PBS, and incubated overnight at 4°C. Saturation was then performed by adding 250 µl of PBS containing 1% BSA and 0.05% tween 20 per well for 90 min at room temperature. Four washes were performed using 200 µl of PBS, 0.05% tween 20 (PBS-T). C1q (10 µg/ml in PBS-T) was added and incubated at room temperature for 90 min. After four washes, bound C1q was detected by successive incubations with an anti-C1q rabbit polyclonal antibody (1:1,000 dilution) and a horseradish peroxidase-conjugated anti-rabbit antibody (Sigma) (1:20,000 dilution). After extensive washes with PBS-T, 100 µl of tetramethylbenzidine solution (Tebu-Bio) was added to each well. The reaction was stopped by adding 100 µl of 1 M H_2_SO_4_ and the optical densiy at 450 nm of each well was measured using an ELISA plate reader (FLUOstar Optima; BMG Labtech).

### Analysis of Small-Angle X-ray Scattering (SAXS)

Small-angle X-ray scattering experiments were performed at the European Synchrotron Radiation Facility (ESRF, Grenoble) beamline BM29, using a Pilatus 1M detector, and an X-ray energy of 12.5 keV. A gel filtration was performed immediately prior to the experiment using a S200 10/300 column (GE Healthcare), 10 mM TRIS, 150 mM NaCl, pH 8.0 in the presence of 1 mM dithiothreitol (DTT) to protect the sample from radiation damage and limit the aggregation. The distance between detector and sample was 2.87 m, covering a q-range of 0.025–5/nm and the sample temperature was set to 4°C. For each dataset, 10 measurements of 1 s exposure were recorded on a 50 µl sample injected into the capillary. Three concentrations of the CCP24-25 fragment were used (serial dilutions: 5, 2.5, 1.25 mg/ml). Images were radially integrated, averaged, and buffer subtracted using the beamline data processing pipeline (31). As some aggregation was observed in the highest concentration even in the presence of 1 mM DTT, the final SAXS curve has been obtained by merging the high angle and low angle regions from the 5 and 1.25 mg/ml, respectively, using the PRIMUS software from the ATSAS package (32). Low resolution *ab initio* envelopes were calculated using DAMMIF and also GASBOR, to make use of good data quality in the high angle region. An intitial model of CCP24-25 was obtained by homology modeling using the server ALLOSMOD (33) and the crystal structure of the CRRY complement receptor (2xrb) as starting template. Two N-glycans were modeled at positions Asn 1534 and Asn 1540 according to the prediction of the NetNGlyc server.^2^ The program CORAL was used to fit the SAXS data by defining two rigid bodies (the CCP24 and CCP25 modules) and two flexible regions (the linker between the modules and the C-terminal His-tag).

## Results

### The CR1 CCP22-30 Fragment Efficiently Binds C1q

**Figure 1:**
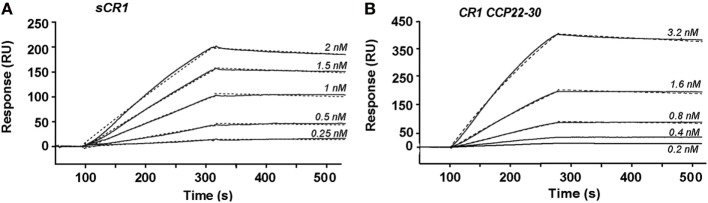
Kinetic analysis of the interaction of C1q with immobilized soluble CR1 (sCR1) and CR1 CCP22-30. C1q was injected at the indicated concentrations over immobilized sCR1 (9,500 RU) **(A)** and CCP22-30 (1,500 RU) **(B)** in 50 mM triethanolamine-HCl (TEA), 145 mM NaCl, 1 mM CaCl_2_, 0.005% surfactant P20, pH 7.4. Fits obtained by a global fitting of the data to a Langmuir 1:1 binding model are shown in dotted lines. The results shown are representative of three experiments (see Table 1).

The CR1 CCP22-30 fragment was originally produced in insect cells as detailed in Ref. (26). Its interaction properties with C1q were analyzed by SPR spectroscopy and compared with those of sCR1. The slight sigmoidal shape of the curves in the association phase and the slow dissociation (Figure 1) suggest a complex interaction model involving multivalent interaction. However, the binding curves could be fitted using a 1:1 Langmuir model with satisfactory Chi^2^ values (around 7) for both sCR1 and the CR1 CCP22-30 fragment. These results show that C1q binds to both sCR1 and CR1 CCP22-30 with comparable association and dissociation constants (*k*_a_ and *k*_d_) resulting in apparent equilibrium dissociation constants (*K*_D_s) in the same nanomolar range of 0.49 and 0.26 nM, respectively (Table 1). This indicates that the CCP22-30 region of CR1 is sufficient for efficient C1q binding and most probably contains the main CR1 interaction sites for C1q.

**Table 1:** Kinetic and equilibrium dissociation constants for the binding of C1q to immobilized soluble CR1 (sCR1) and CR1 CCP22-30.

	*k*_a_ (M^−1^ s^−1^)	*k*_d_ (s^−1^)	*K*_D_ (nM)	*n*^a^
Immobilized sCR1	1.30 ± 0.22 × 10^6^	6.60 ± 1.47 × 10^−4^	0.49 ± 0.04	3
Immobilized CR1 CCP22-30	1.63 ± 0.70 × 10^6^	4.26 ± 2.27 × 10^−4^	0.26 ± 0.07	3

*Values are expressed as means ± SE*.

*^a^Number of separate experiments on different sensorchips*.

### Location of the C1q-Binding Site in CR1 CCP22-30 Using Deletion CR1 Variants

**Figure 2:**
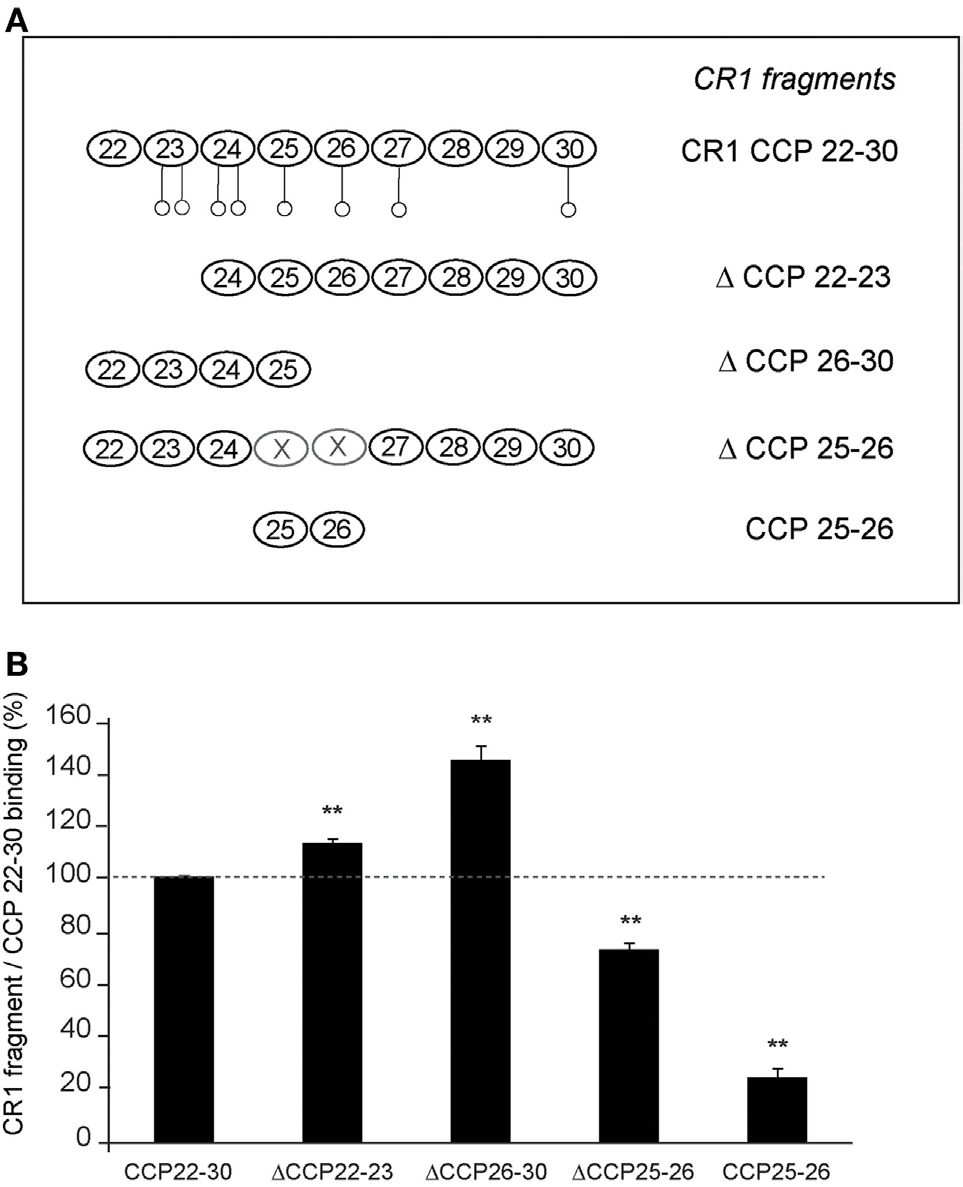
Localization of the C1q binding sites in CR1 CCP22-30 using deletion fragments. **(A)** Schematic view of the different fragments of CR1 CCP22-30. Potential N-glycosylations are represented by open circles ○. **(B)** Interaction of C1q with immobilized CR1 CCP22-30 fragments analyzed by ELISA. C1q (10 µg/ml in PBS) was added to microtiter plate coated with 3.4 pmol of each CCP22-30 fragment as described in Section “Materials and Methods.” After 1.5 h incubation at room temperature, bound C1q was detected with rabbit antibodies recognizing C1q and HRP secondary antibodies. Data are presented as the mean ± SE of four individual experiments. (**), Student’s *t*-test values *p* < 0.01 of C1q binding to each fragment compared to CCP22-30.

With a view to locate more precisely the modules of the CR1 CCP22-30 region involved in C1q interaction, we used various CR1 CCP22-30 fragments represented schematically in Figure 2A. Solid-phase binding assays with immobilized CR1 variants, lead to the results summarized in Figure 2B. Fragments ΔCCP22-23 and ΔCCP26-30 were still able to bind to C1q and their binding capacities were even increased by comparison with CR1 CCP22-30. These results indicate that modules CCP24-30 and CCP22-25 contain the determinants required for C1q binding, suggesting the involvement of the CCP24-25 segment common to both variants. The higher binding capacity of both variants could likely be explained by a better accessibility of the C1q binding site, expecially in the shorter fragment ΔCCP26-30. Furthermore, the importance of CCP24 and CCP25 in C1q binding was confirmed by the interaction capacities of both fragment ΔCCP25-26 and the bimodular fragment CCP25-26. Indeed, the decreased C1q binding observed with fragment ΔCCP25-26 (27%), where CR1 CCP25-26 are missing, supports the implication of CCP25 in C1q binding. In addition, the low binding (24%) of the CCP25-26 fragment points out the importance of CCP24 in C1q binding. Overall, these CR1 deletion variants highlight the importance of the CR1 module pair CCP24-CCP25 in C1q interaction.

### C1q and MBL Both Interact with CR1 in the Same CCP24-25 Region

**Figure 3:**
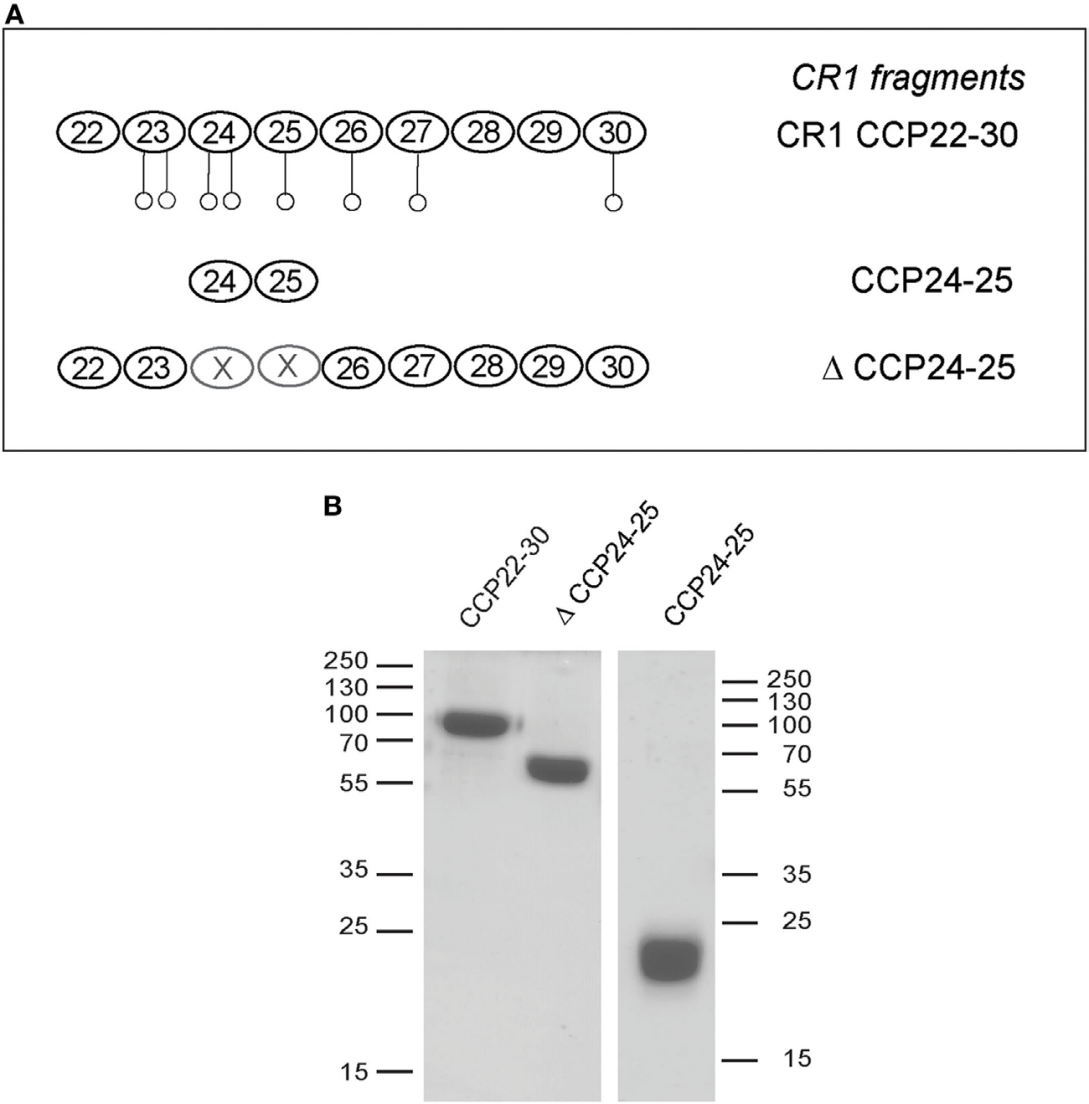
Complement receptor type 1 (CR1) variants produced to study the CCP24-25 module pair interaction properties. **(A)** Schematic view of CCP24-25 variants produced in eukaryotic cells. Potential N-glycosylations are represented by open circles ○. **(B)** SDS-PAGE analysis of 4 µg of the CR1variants under reducing conditions. The positions of the molecular weight markers (expressed in kilodaltons) are indicated. *A full scan of the original gel is provided in Figure S1 in Supplementary Material*.

**Figure 4:**
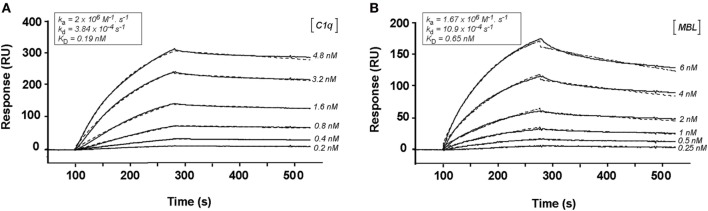
Kinetic analysis of the interaction of C1q and mannose-binding lectin (MBL) with CR1 CCP24-25. C1q **(A)** and MBL **(B)**, at indicated concentrations, were injected in 50 mM triethanolamine-HCl (TEA), 145 mM NaCl, 0.05% surfactant P20, pH 7.4, on immobilized CR1 CCP24-25 (1,000 RU). The buffer was supplemented with 3 mM EDTA for MBL binding. Fits are shown as dotted lines and were obtained by global fitting of the data using a 1:1 Langmuir-binding model. The kinetic constants obtained are framed on the top of each sensorgramm.

**Figure 5:**
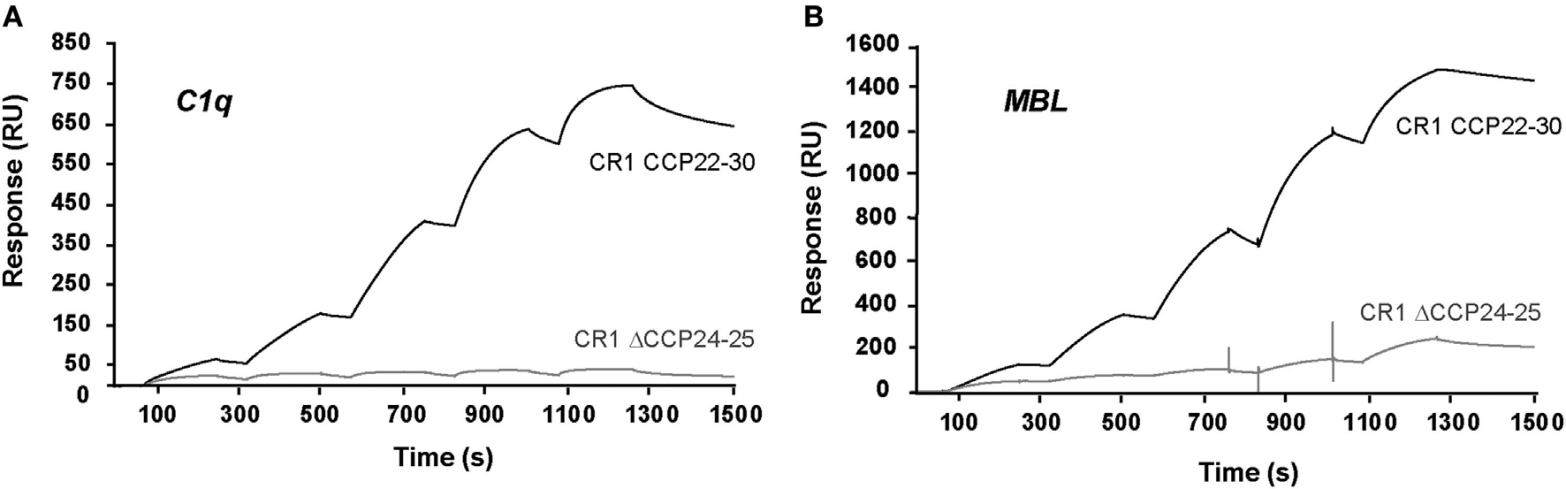
Interaction of C1q and mannose-binding lectin (MBL) with immobilized CR1 CCP22-30 and CR1 ΔCCP24-25. The binding curves were obtained in single cycle mode by injecting increasing concentrations of C1q **(A)** or MBL **(B)** on immobilized CR1 CCP22-30 (3,000 RU) or CR1 ΔCCP24-25 (2,200 RU). C1q (0.25, 0.5, 1, 2, and 4 nM) was injected at 20 µl/min for 180 s in 50 mM triethanolamine-HCl (TEA), 150 mM NaCl, 1 mM CaCl_2_, 0.05% surfractant P20, pH 7.4. MBL (1, 2, 4, 8, and 16 nM) was injected in the same conditions except that the buffer contained 3 mM EDTA instead of CaCl_2_.

We have shown previously using a similar approach that the binding site of CR1 for MBL is most probably located as shown here for C1q, in the same pair of modules CCP24-25. However, none of the fragments used focused exclusively on the CCP24-25 modules. For that reason, we decided to produce two additional CR1 CCP22-30 fragments, one with a deletion of CCP24-25 called ΔCCP24-25 and the bimodular fragment CCP24-25 (Figure 3A). In order to improve the purification of CR1 CCP22-30 and ΔCCP24-25, a 6His-tag was introduced at the C-terminal end of the fragments as explained in Section “Materials and Methods.” No interaction difference was noticed depending on the cellular expression system used (insect cells or mammalian FreeStyle 293-F cells) or on the presence or absence of the 6His-Tag (26). These observations imply that the sialic acid moiety present on the extremities of N-linked glycans in protein expressed in mammalian cells but not on insect cells has no impact on CR1 interaction properties with defense collagens. Both CR1 CCP22-30 and ΔCCP24-25 variants were purified using a one-step nickel-affinity chromatography. The mean yields of purified CCP24-25 and ΔCCP24-25 fragments from 1 l of expression supernatant were, respectively, around 3 and 4.5 mg. As observed by SDS-PAGE analysis (Figure 3B), each fragment was pure and migrated as a single band of apparent molecular weight in reducing conditions of, respectively, around 60 (ΔCCP24-25) and 21 kDa (CCP24-25). Mass spectrometry analysis yielded heterogeneous peaks centered on 57,034 ± 60 and 18,500 ± 20 Da for ΔCCP24-25 and CCP24-25, respectively, accounting for the polypeptide chains (49,918 and 15,388 Da) plus extra masses (7,116 and 3,112 Da) corresponding to N-linked carbohydrates. The interaction properties of both fragments for C1q and MBL were then assessed using SPR spectroscopy (Figure 4). CR1 CCP24-25 interacted with C1q and MBL with high affinity, as reflected by *K*_D_ values in the nM range (0.19 and 0.65 nM, respectively), the complex with C1q being slightly more stable than the complex with MBL, as indicated by a 3.5-fold lower dissociation rate constant (3.84 10^−4^ s^−1^ versus 1.09 10^−3^ s^−1^). The specificity of C1q and MBL binding for CR1 CCP24-25 was then controlled using the deletion fragment ΔCCP24-25. As shown in Figure 5, deletion of CCP24-25 abolished the interaction with C1q and strongly decreased binding of MBL. Taken together, these results unambigously restrain the main interaction sites of CR1 for both MBL and C1q to the same pair of modules CCP24-25.

### The CCP24-25 Fragment Pair Adopts an Extended Conformation in Solution

**Figure 6:**
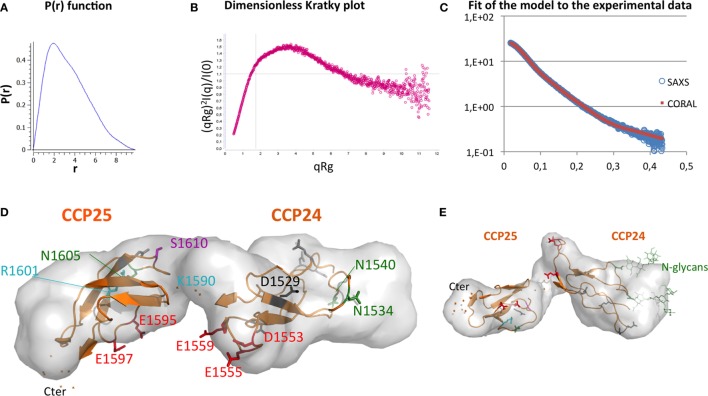
Elongated shape of CCP24-25, associated to a possible interpretative model. **(A)** Small-angle X-ray scattering (SAXS) pair distance distribution, **(B)** SAXS dimensionless Kratky plot, which shows elongation and remaining flexibility. **(C)** Fit of the model [shown in panels **(D,E)**] to the experimental data. **(D)** Top and **(E)** side views of an *ab initio* envelope computed with GASBOR. A Coral-derived model of CCP24-25 is shown inside to illustrate how the shape corresponds to two CCP modules. This interpretative model is used to illustrate the positions on each module of glycosylation sites (green), rs 4844609 SNP (magenta), Knops groups variants (cyan), acidic clusters (red) initially suggested as potential MBL binding sites, and the other acidic residues (gray and black). D1529 is the homologous counterpart of D1076 in CR1 CCP17 (black). The two flexible carbohydrates included in the model are only illustrated in the side view.

Small-angle X-ray scattering analysis has been performed on the CCP24-25 fragment produced in insect cells at the ESRF BM29 beamline, as described in Section “Materials and Methods.” The global parameters derived from the SAXS data (Table 2) characterize the CCP24-25 fragment as a monomeric protein having an Rg of about 2.7 nm. The pair distribution P(r) curve shows the typical aspect of an elongated multidomain protein with a Dmax of ~9.8 nm (Figure 6A). Kratky analysis clearly shows flexibility with a maximum at q*Rg ~ 3.5 which is significantly higher than the theoretical value (√3) that is characteristic of well folded, globular proteins (34) (Figure 6B). Also the curve does not tend to 0 at high q*Rg indicating high local flexibility that could be mainly attributed to the presence of the glycans, but also to the C-terminal histidine tag and to the semi-flexible linker. The overall *ab initio* envelopes, calculated by the GASBOR software, show two elongated blobs with a global boomerang shape (Figures 6D–E). Temptatively, one could attribute the larger blob to the CCP24 which is slightly bigger in size and also bears two N-glycans. The molecular weight estimate from the *ab initio* envelope is ~18.1 kDa which is in good agreement with the experimental value (18.5 kDa). Rigid body modeling using the program CORAL resulted in an extended conformation with the bend point located between the two CCP modules and a good fit to the experimental data (χ = 1.2) that could be superposed on the *ab initio* envelope (Figures 6C–E).

**Table 2:** Overall small-angle X-ray scattering experimental parameters for the CCP24-25 fragment.

Conc (mg/ml)	Rg Guinier (nm)	Q*Rg limits	I(0)	Rg Gnom (nm)	Dmax (nm)	MW Gnom^a^	MW Dammif^a^
1.25	2.67 ± 0.19	0.47–1.19	27.4 ± 0.05	2.74	9.8	15.8	–
2.5	2.78 ± 0.12	0.52–1.12	28.7 ± 0.04	2.78	9.8	16.4	–
5.0	2.76 ± 0.53^b^	0.92–1.24	29.2 ± 0.07	2.84	9.8	16.4	–
Merged	2.67 ± 0.2	0.47–1.19	27.4 ± 0.04	2.74	9.8	15.6	18.1

*^a^The MW Gnom is calculated from the Porod volume. The MW Dammif is calculated from the ab initio envelope*.

*^b^Aggregation*.

### Location of the CR1-Binding Sites within C1q

**Figure 7:**
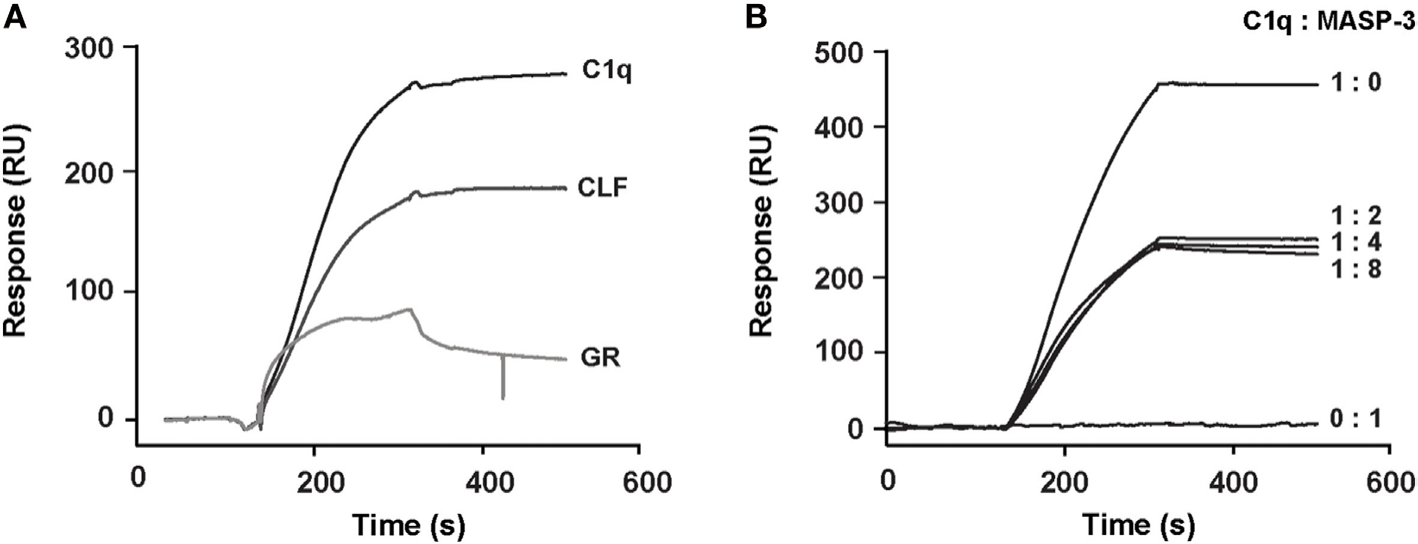
Localization of the binding site of soluble CR1 on C1q. **(A)** Comparative binding of C1q and its CLF and GR on complement receptor type 1 (CR1) CCP22-30 analyzed by surface plasmon resonance (SPR). The CR1 CCP22-30 fragment was immobilized on CM5 sensor chips (4,500 RU) and 2 nM of C1q or CLF and 12 nM of GR were injected over the surfaces as described in Section “Materials and Methods.” **(B)** MBL-associated serine protease (MASP)-3 competition of the binding of C1q to CR1 CCP22-30 analyzed by SPR. C1q (2 nM) was incubated at room temperature for 20 min in the absence or presence of recombinant MASP-3 at indicated molar ratios and injected over immobilized CR1 CCP22-30 (4,500 RU). No binding was observed when MASP-3 alone (20 nM) was injected.

In order to identify the region of C1q that is involved in CR1 interaction, the comparative binding of C1q collagen stalks (CLF), C1q globular heads (GR), and whole C1q to CR1 CCP22-30 was performed by SPR. The results shown in Figure 7A indicate that CR1 mostly interacts with C1q CLF although binding to C1q GR is also observed. Moreover, the competition with MASP-3, used as a competitor for the C1r/C1s serine protease interaction site on C1q (35) (Figure 7B), confirms that there is indeed a binding site on the collagen stalks of C1q likely located on or in close proximity of the C1r/C1s interaction site. Interestingly, the inhibition is not complete when MASP-3 occupies all the potential C1q sites (C1q:MASP-3 dimer molar ratio of 1:2) and does not decrease when MASP-3 is added in excess (1:4 and 1:8 ratios). The remaining interaction observed with maximal MASP-3 competition (around 50%) might be due to the contribution of other binding sites on the CLF and/or on the globular regions of C1q.

## Discussion

Previous studies have reported that CR1 is a binding platform for complement opsonins C3b, C4b and defense collagens, such as C1q, MBL, and ficolin-2 (15, 26, 36–39). The present work, based on a dissection strategy, highlights that the interaction region of CR1 with C1q and MBL on CR1-LHR-D is restricted to only two CCP modules, CCP24 and CCP25. We first confirmed in this study that CR1 fragment CCP22-30 encompassing the LHR-D plus two additional modules (CCP29-30) contains a site of interaction for C1q. This was previously shown using lysates of transfected Chinese hamster ovary cells (15) but is demonstrated here using purified recombinant CR1 fragments expressed in eukaryotic cells. For all CR1 fragments produced, the interaction could be only measured by SPR with immobilized CR1 as it was also the case for MBL (26). The explanation for that observation is probably due to the organization of CR1 on the cell membrane into clusters that would require a similar molecule presentation *in vitro* to allow the right positioning of multiple sites suitable for the binding of defense collagens (40, 41). The affinity of C1q for CR1 obtained by SPR was comparable to that previously found for MBL interaction (26) with *K*_D_s of 0.49 nM (MBL 0.76 nM) for sCR1 and 0.26 nM (MBL 0.37 nM) for CR1 CCP22-30. For both C1q and MBL, the CCP22-30 fragment had an affinity that was slightly higher than that of full-length sCR1, indicating that the site of interaction for the defense collagens is located mainly in this region with no or little implication of other LHRs. This is in accordance with the additive interaction of C3b, C4b, and C1q for CR1 observed by Tas et al. (16) and more recently with the results of Ref. (42) that the CR1 CCP15-25 fragment is able to interact with C1q. Using a panel of deletion fragments of CR1 CCP22-30 (26), we get an indirect indication of the CCP24-25 implication in CR1 interaction with C1q, as shown also for MBL and ficolin-2. The present work aimed at going deeper into the characterization of the interaction of CR1 CCP24-25 with C1q and MBL. Direct evidence for the involvement of the two CCP24 and CCP25 modules was provided by the production of two supplementary fragments, one consisting of the CCP24-25 bimodular fragment, and the second one corresponding to the CR1 CCP22-30 fragment deleted from the two CCP24-25 modules. The CCP24-25 bimodular fragment was shown to interact equally with C1q and MBL. In addition, the CCP24-25 specificity was also clearly demonstrated by binding assays on the truncated fragment ΔCCP24-25 that showed no binding for C1q and negligible binding to MBL compared to the entire CR1 CCP22-30. Taken together, these results clearly assign the binding specificity of CR1 for defense collagens C1q and MBL to its CCP24-25 module pair.

Although the whole CR1 molecule shows a partially folded back solution structure (43), we obtained SAXS data showing an extended conformation of the CCP24-25 module pair in solution, which is fully consistent with the elongated conformation mainly observed for other complement regulators interactions (25). From these data, we could propose an interpretative model (illustrated in Figure 6D), which can be used as a support for discussion. Two clusters of acidic residues were initially suggested as possible common interaction sites for the conserved lysine residues on the collagen stalks of MBL and ficolin-2 (26). They are shown in red in Figure 6D and correspond to Asp 1553, Glu, 1555 and Glu 1559 in CCP24 and Glu 1595 and Glu 1597 in CCP25. These acidic residues were selected because: (i) the interaction of CR1 with C1q or MBL is highly sensitive to ionic strength (15, 39); (ii) MASP-3 can compete with the interaction between CR1 and the defense collagens (MBL, ficolin-2, C1q), which suggests a common binding to the conserved lysine residues in the collagen stalks of these proteins (29, 35, 44); (iii) the proposed clusters are unique to the LHR-D, including at least one acidic residue which has no counterpart in the other LHR regions. We have tested the charge effect of these acidic residues by mild mutations into their corresponding amide residue (Asp/Asn and Glu/Gln), thus preserving their overall shape but not their charge. Unfortunately, the mutations do not significantly affect the binding of CR1 to MBL and C1q (data not shown). From this negative result, it can be deduced at least that the charge effect of these acidic clusters is not essential or might be compensated by the remaining acidic residues (5 on CCP24 and 1 in CCP25 in gray or black in Figure 6D). A recent study on CR1 isolated from homozygous and heterozygous donors for the rs 4844609 SNP (resulting in the change of Ser 1610 to Thr in module CCP25, in magenta in Figure 6D) showed a small but significant increase in C1q binding compared to CR1 without this SNP (45). Although we could not reproduce this observation with a CR1CCP22-30 Ser 1610 Thr mutant produced in our laboratory, this might indicate that the C1q-binding site could be located nearby the Ser1610. On the other end, Knops blood groups variants are also in close proximity to the mutated acidic residues and it could be noticed that charge differences on Knops group variants (cyan) (42) have no effect on C1q binding. Moreover, sialic acids at the glycosylation extremities (glycosylation sites in dark green in Figure 6D) do not influence C1q binding either. Apparently, this is not a simple question and further investigations will be required to precisely define the collagen binding-site residues. Among possible directions, we might consider the hypothesis that the charge interaction involves an acidic residue which is conserved in all LHR regions, the selectivity for the collagen-like partners deriving from the evolution of its surrounding residues. Following this altenative hypothesis, one interesting candidate would be Asp1529, because Asp1076, its homologous counterpart in CCP17, provides the main ionic interaction of CCP17 with C3b, in direct interaction with C3b Arg1310 [PDB 5O9 (25)]. Overall, this might suggest that C1q interacts in between the two modules, possibly on the upper face (Figure 6D) including this Asp 1529, Ser 1610 and four other non-mutated acidic residues (in gray).

Finally, the other goal of the present work was to identify the regions on C1q that are responsible for the interaction with CR1. It was previously shown that C1q collagen tails interact with sCR1 and can also mediate C1q binding to erythrocytes CR1 (15, 16). We confirm the importance of C1q CLF in CR1 interaction using SPR but in addition our results also bring out the potential implication of subsidiary sites on C1q for CR1 binding. This conclusion arises from two different approaches: (i) the interaction of C1q CLF and C1q globular heads with immobilized CR1 CCP22-30 by SPR showing that there is a contribution of both C1q regions in CR1 binding (ii) the competition with MASP-3 that does not completely abolish C1q binding to CR1. Moreover, mutating the protease-binding site (LysB61A, LysC58A) in C1q (35) reduces without abolishing, the interaction with CR1 (Figure S2 in Supplementary Material). Overall these results point out that there are additionnal sites on C1q for CR1 interaction most probably located on C1q globular heads. In this respect it can not be excluded that the short overlapping sequences common to the CLF and the GR, due to the generation of both fragments by limited proteolysis of C1q (residues A85-97, B81-97, C78-94) might contribute to CR1 interactions (46, 47). In the case of MBL, our previous studies showed unambigously that the interaction with CR1 involves only its collagen stalks (26). However, eventhough MBL and C1q are homologous proteins, the case of C1q is more complex and might be of physiological relevance. Effectively, C1q has been reported to serve as a bridging molecule for membrane receptors involved in immune tolerance. This is the case for C1q interaction with CD91/CRT involved in the clearance of apoptotic cells (48) and also, as described more recently, for the inhibitory immunoreceptors implicating partnering of C1q/LAIR-1/RAGE and HMGB1 (49) or C1q/LAIR-1/CD33 (13). In all these bridging interactions, C1q engages both its collagen tails and globular heads. One can, therefore, postulate that since CR1 is a large receptor clustered on the cell membrane, it can present a multisite platform for an efficient interaction with C1q.

## Author Contributions

VR, NT, and CG designed the study; MJ, GC, GF, and IB performed the research; MJ, GC, VR, CG, and NT analyzed the data; VR, NT, and CG wrote the manuscript; all authors revised and approved the final version of the manuscript.

## Conflict of Interest Statement

The authors declare that the research was conducted in the absence of any commercial or financial relationships that could be construed as a potential conflict of interest.

## Footnotes

^1^http://web.expasy.org/protparam/.

^2^http://www.cbs.dtu.dk/services/NetNGlyc/.

## Abbreviations

GR, globular region of C1q; CLF, collagenic stalks of C1q; CR1, complement receptor type 1; sCR1, soluble CR1; CCP, complement control protein repeat; LRP1, LDL receptor related protein 1; RAGE, receptor for advanced glycation end products; Siglec-3, sialic-acid-binding immunoglobulin-like lectin-3; LAIR-1, leukocyte associated immunoglobulin-like receptor 1; CRT, calreticulin; MBL, mannose-binding lectin; MASP, MBL-associated serine protease; SPR, surface plasmon resonance; RU, resonance unit; TEA, triethanolamine-HCl; SAXS, small-angle X-ray scattering; ESRF, European Synchrotron Radiation Facility.

## References

[1] Bobak DA Washburn RG Frank MM. C1q enhances the phagocytosis of *Cryptococcus neoformans* blastospores by human monocytes. J Immunol (1988) 141:592–7. 3290342

[2] Webster SD Galvan MD Ferran E Garzon-Rodriguez W Glabe CG Tenner AJ. Antibody-mediated phagocytosis of the amyloid beta-peptide in microglia is differentially modulated by C1q. J Immunol (2001) 166:7496–503. 10.4049/jimmunol.166.12.7496 11390503

[3] Nayak A Pednekar L Reid KB Kishore U. Complement and non-complement activating functions of C1q: a prototypical innate immune molecule. Innate Immun (2012) 18:350–63. 10.1177/1753425910396252 21450789

[4] Bohlson SS O’Conner SD Hulsebus HJ Ho M-M Fraser DA. Complement, C1q, and C1q-related molecules regulate macrophage polarization. Front Immunol (2014) 5:402. 10.3389/fimmu.2014.00402 25191325 PMC4139736

[5] Gaboriaud C Frachet P Thielens NM Arlaud GJ. The human C1q globular domain: structure and recognition of non-immune self ligands. Front Immunol (2012) 2:92. 10.3389/fimmu.2011.00092 22566881 PMC3342031

[6] Kouser L Madhukaran SP Shastri A Saraon A Ferluga J Al-Mozaini M Emerging and novel functions of complement protein C1q. Front Immunol (2015) 6:317. 10.3389/fimmu.2015.00317 26175731 PMC4484229

[7] Bohlson SS Fraser DA Tenner AJ. Complement proteins C1q and MBL are pattern recognition molecules that signal immediate and long-term protective immune functions. Mol Immunol (2007) 44:33–43. 10.1016/j.molimm.2006.06.021 16908067

[8] Degn SE Thiel S. Humoral pattern recognition and the complement system. Scand J Immunol (2013) 78:181–93. 10.1111/sji.12070 23672641

[9] Ghebrehiwet B Hosszu KK Valentino A Ji Y Peerschke EIB Monocyte expressed macromolecular C1 and C1q receptors as molecular sensors of danger: implications in SLE. Front Immunol (2014) 5:278 10.3389/fimmu.2014.00278 25018754 PMC4071343

[10] Thielens NM Tedesco F Bohlson SS Gaboriaud C Tenner AJ. C1q: a fresh look upon an old molecule. Mol Immunol (2017) 89:73–83. 10.1016/j.molimm.2017.05.025 28601358 PMC5582005

[11] Paidassi H Tacnet-Delorme P Arlaud GJ Frachet P. How phagocytes track down and respond to apoptotic cells. Crit Rev Immunol (2009) 29:111–30. 10.1615/CritRevImmunol.v29.i2.20 19496743

[12] Son M Santiago-Schwarz F Al-Abed Y Diamond B. C1q limits dendritic cell differentiation and activation by engaging LAIR-1. Proc Natl Acad Sci U S A (2012) 109:E3160–7. 10.1073/pnas.1212753109 23093673 PMC3503216

[13] Son M Diamond B Volpe BT Aranow CB Mackay MC Santiago-Schwarz F. Evidence for C1q-mediated crosslinking of CD33/LAIR-1 inhibitory immunoreceptors and biological control of CD33/LAIR-1 expression. Sci Rep (2017) 7:270. 10.1038/s41598-017-00290-w 28325905 PMC5412647

[14] Eggleton P Tenner AJ Reid KBM C1q receptors. Clin Exp Immunol (2000) 120:406–12. 10.1046/j.1365-2249.2000.01218.x 10844516 PMC1905565

[15] Klickstein LB Barbashov SF Liu T Jack RM Nicholson-Weller A. Complement receptor type 1 (CR1, CD35) is a receptor for C1q. Immunity (1997) 7:345–55. 10.1016/S1074-7613(00)80356-8 9324355

[16] Tas SW Klickstein LB Barbashov SF Nicholson-Weller A. C1q and C4b bind simultaneously to CR1 and additively support erythrocyte adhesion. J Immunol (1999) 163:5056–63. 10528211

[17] Fearon D. Identification of the membrane glycoprotein that is the C3b receptor of the human erythrocyte, polymorphonuclear leukocyte, B lymphocyte, and monocyte. J Exp Med (1980) 152:20–30. 10.1084/jem.152.1.20 6967510 PMC2185895

[18] Ross GD Yount WJ Walport MJ Winfield JB Parker CJ Fuller CR Disease-associated loss of erythrocyte complement receptors (CR1, C3b receptors) in patients with systemic lupus erythematosus and other diseases involving autoantibodies and/or complement activation. J Immunol (1985) 135:2005–14. 4020137

[19] Fearon DT Klickstein LB Wong WW Wilson JG Moore FD Weis JJ Immunoregulatory functions of complement: structural and functional studies of complement receptor type 1 (CR1; CD35) and type 2 (CR2; CD21). Prog Clin Biol Res (1989) 297:211–20. 2531419

[20] Birmingham DJ Hebert LA. CR1 and CR1-like: the primate immune adherence receptors. Immunol Rev (2001) 180:100–11. 10.1034/j.1600-065X.2001.1800109.x 11414352

[21] Java A Liszewski MK Hourcade DE Zhang F Atkinson JP. Role of complement receptor 1 (CR1; CD35) on epithelial cells: a model for understanding complement-mediated damage in the kidney. Mol Immunol (2015) 67:584–95. 10.1016/j.molimm.2015.07.016 26260209 PMC4565762

[22] Krych-Goldberg M Hauhart RE Subramanian VB Yurcisin BM Crimmins DL Hourcade DE Decay accelerating activity of complement receptor type 1 (CD35). Two active sites are required for dissociating C5 convertases. J Biol Chem (1999) 274:31160–8. 10.1074/jbc.274.44.31160 10531307

[23] Krych-Goldberg M Atkinson JP. Structure-function relationships of complement receptor type 1. Immunol Rev (2001) 180:112–22. 10.1034/j.1600-065X.2001.1800110.x 11414353

[24] Smith BO Mallin RL Krych-Goldberg M Wang X Hauhart RE Bromek K Structure of the C3b binding site of CR1 (CD35), the immune adherence receptor. Cell (2002) 108:769–80. 10.1016/S0092-8674(02)00672-4 11955431

[25] Forneris F Wu J Xue X Ricklin D Lin Z Sfyroera G Regulators of complement activity mediate inhibitory mechanisms through a common C3b-binding mode. EMBO J (2016) 35:1133–49. 10.15252/embj.201593673 27013439 PMC4868954

[26] Jacquet M Lacroix M Ancelet S Gout E Gaboriaud C Thielens NM Deciphering complement receptor type 1 interactions with recognition proteins of the lectin complement pathway. J Immunol (2013) 190:3721–31. 10.4049/jimmunol.1202451 23460739

[27] Arlaud GJ Sim RB Duplaa AM Colomb MG Differential elution of Clq, Clr and Cls from human Cl bound to immune aggregates. Use in the rapid purification of Cl subcomponents. Mol Immunol (1979) 16:445–50. 10.1016/0161-5890(79)90069-5 40870

[28] Tacnet-Delorme P Chevallier S Arlaud GJ Amyloid fibrils activate the C1 complex of complement under physiological conditions: evidence for a binding site for A on the C1q globular regions. J Immunol (2001) 167:6374–81. 10.4049/jimmunol.167.11.6374 11714802

[29] Teillet F Lacroix M Thiel S Weilguny D Agger T Arlaud GJ Identification of the site of human Mannan-binding lectin involved in the interaction with its partner serine proteases: the essential role of Lys55. J Immunol (2007) 178:5710–6. 10.4049/jimmunol.178.9.5710 17442954

[30] Wang W Malcolm BA. Two-stage PCR protocol allowing introduction of multiple mutations, deletions and insertions using QuikChange Site-Directed Mutagenesis. Biotechniques (1999) 26:680–2. 10343905 10.2144/99264st03

[31] Brennich ME Kieffer J Bonamis G De Maria Antolinos A Hutin S Pernot P Online data analysis at the ESRF bioSAXS beamline, BM29. J Appl Crystallogr (2016) 49:203–12. 10.1107/S1600576715024462

[32] Franke D Petoukhov MV Konarev PV Panjkovich A Tuukkanen A Mertens HDT ATSAS 2.8: a comprehensive data analysis suite for small-angle scattering from macromolecular solutions. J Appl Crystallogr (2017) 50:1212–25. 10.1107/S1600576717007786 28808438 PMC5541357

[33] Weinkam P Chen YC Pons J Sali A. Impact of mutations on the allosteric conformational equilibrium. J Mol Biol (2013) 425:647–61. 10.1016/j.jmb.2012.11.041 23228330 PMC3557769

[34] Durand D Vivès C Cannella D Pérez J Pebay-Peyroula E Vachette P NADPH oxidase activator p67(phox) behaves in solution as a multidomain protein with semi-flexible linkers. J Struct Biol (2010) 169:45–53. 10.1016/j.jsb.2009.08.009 19723583

[35] Bally I Ancelet S Moriscot C Gonnet F Mantovani A Daniel R Expression of recombinant human complement C1q allows identification of the C1r/C1s-binding sites. Proc Natl Acad Sci U S A (2013) 110:8650–5. 10.1073/pnas.1304894110 23650384 PMC3666734

[36] Klickstein LB Bartow TJ Miletic V Rabson LD Smith JA Fearon DT. Identification of distinct C3b and C4b recognition sites in the human C3b/C4b receptor (CR1, CD35) by deletion mutagenesis. J Exp Med (1988) 168:1699–717. 10.1084/jem.168.5.1699 2972794 PMC2189104

[37] Makrides SC Scesney SM Ford PJ Evans KS Carson GR Marsh HC. Cell surface expression of the C3b/C4b receptor (CR1) protects Chinese hamster ovary cells from lysis by human complement. J Biol Chem (1992) 267:24754–61. 1447213

[38] Krych M Hourcade D Atkinson JP Sites within the complement C3b/C4b receptor important for the specificity of ligand binding. Proc Natl Acad Sci U S A (1991) 88:4353–7. 10.1073/pnas.88.10.4353 1827918 PMC51657

[39] Ghiran I Barbashov SF Klickstein LB Tas SW Jensenius JC Nicholson-Weller A. Complement receptor 1/CD35 is a receptor for Mannan-binding lectin. J Exp Med (2000) 192:1797–808. 10.1084/jem.192.12.1797 11120776 PMC2213499

[40] Chevalier J Kazatchkine MD. Distribution in clusters of complement receptor type one (CR1) on human erythrocytes. J Immunol (1989) 142:2031–6. 2522131

[41] Lapin ZJ Höppener C Gelbard HA Novotny L. Near-field quantification of complement receptor 1 (CR1/CD35) protein clustering in human erythrocytes. J Neuroimmune Pharmacol (2012) 7:539–43. 10.1007/s11481-012-9346-3 22374251

[42] Tetteh-Quarcoo PB Schmidt CQ Tham W-H Hauhart R Mertens HDT Rowe A Lack of evidence from studies of soluble protein fragments that knops blood group polymorphisms in complement receptor-type 1 are driven by malaria. PLoS One (2012) 7:e34820. 10.1371/journal.pone.0034820 22506052 PMC3323580

[43] Furtado PB Huang CY Ihyembe D Hammond RA Marsh HC Perkins SJ. The partly folded back solution structure arrangement of the 30 SCR domains in human complement receptor type 1 (CR1) permits access to its C3b and C4b ligands. J Mol Biol (2008) 375:102–18. 10.1016/j.jmb.2007.09.085 18028942

[44] Lacroix M Dumestre-Pérard C Schoehn G Houen G Cesbron J-Y Arlaud GJ Residue Lys57 in the collagen-like region of human L-ficolin and its counterpart Lys47 in H-ficolin play a key role in the interaction with the Mannan-binding lectin-associated serine proteases and the collectin receptor calreticulin. J Immunol (2009) 182:456–65. 10.4049/jimmunol.182.1.456 19109177

[45] Fonseca MI Chu S Pierce AL Brubaker WD Hauhart RE Mastroeni D Analysis of the putative role of CR1 in Alzheimer’s disease: genetic association, expression and function. PLoS One (2016) 11:e0149792 10.1371/journal.pone.0149792 26914463 PMC4767815

[46] Reid KBM. Isolation, by partial pepsin digestion, of the three collagen-like regions present in subcomponent Clq of the first component of human complement. Biochem J (1976) 155:5–17. 10.1042/bj1550005 7240 PMC1172796

[47] Gaboriaud C Juanhuix J Gruez A Lacroix M Darnault C Pignol D The crystal structure of the globular head of complement protein C1q provides a basis for its versatile recognition properties. J Biol Chem (2003) 278:46974–82. 10.1074/jbc.M307764200 12960167

[48] Gardai SJ Xiao Y-Q Dickinson M Nick JA Voelker DR Greene KE By binding SIRPα or calreticulin/CD91, lung collectins act as dual function surveillance molecules to suppress or enhance inflammation. Cell (2003) 115:13–23. 10.1016/S0092-8674(03)00758-X 14531999

[49] Son M Porat A He M Suurmond J Santiago-Schwarz F Andersson U C1q and HMGB1 reciprocally regulate human macrophage polarization. Blood (2016) 128:2218–28. 10.1182/blood-2016-05-719757 27683415 PMC5095756

