# Supplementary figures and images for: C1q and Mannose-Binding Lectin Interact with CR1 in the Same Region on CCP24-25 Modules
Source: Front Immunol. 2018 Mar 7;9:453. doi: 10.3389/fimmu.2018.00453 (PMC5845983; doi:10.3389/fimmu.2018.00453)

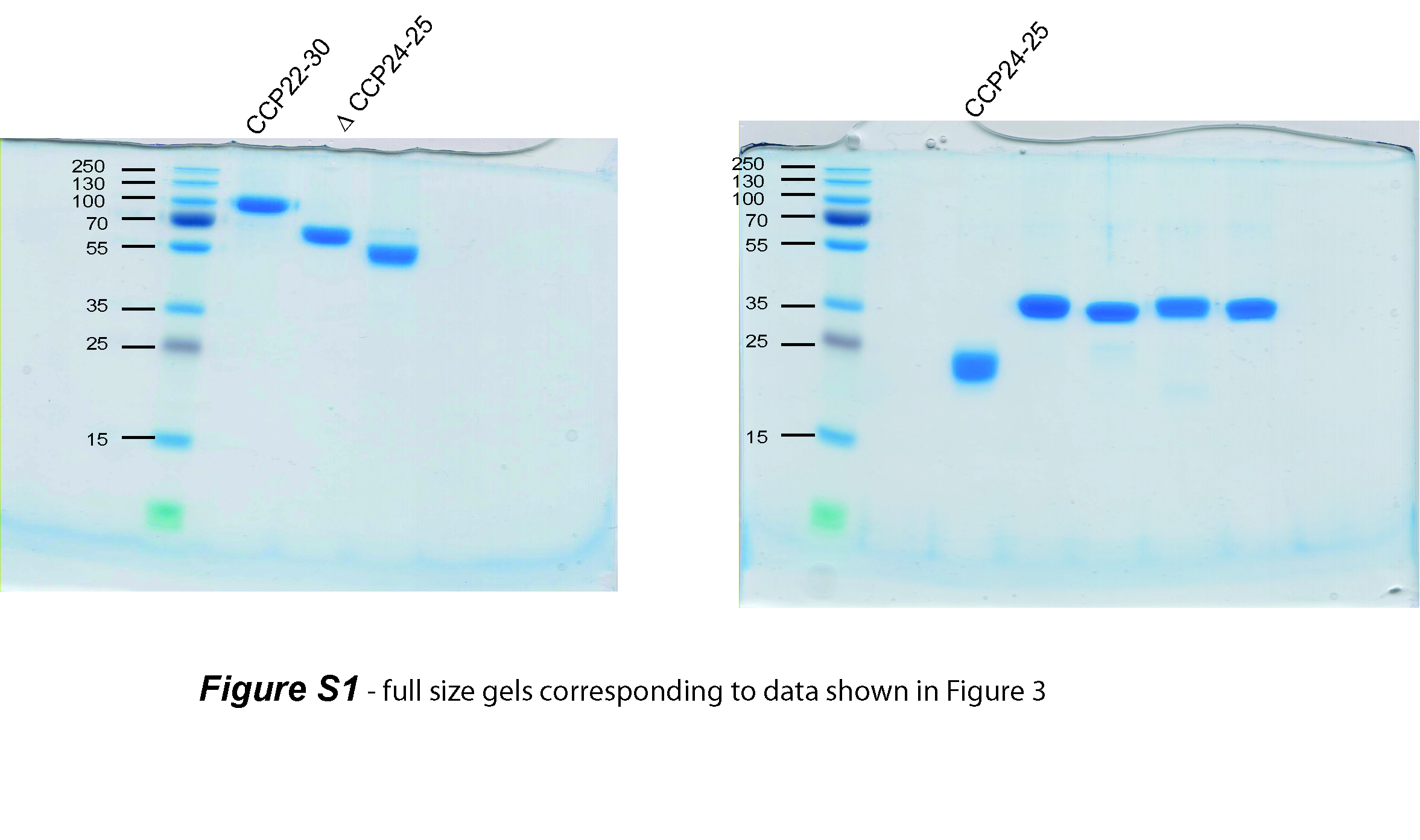

Supplement: Supplementary file 1 [file image_1.tif]

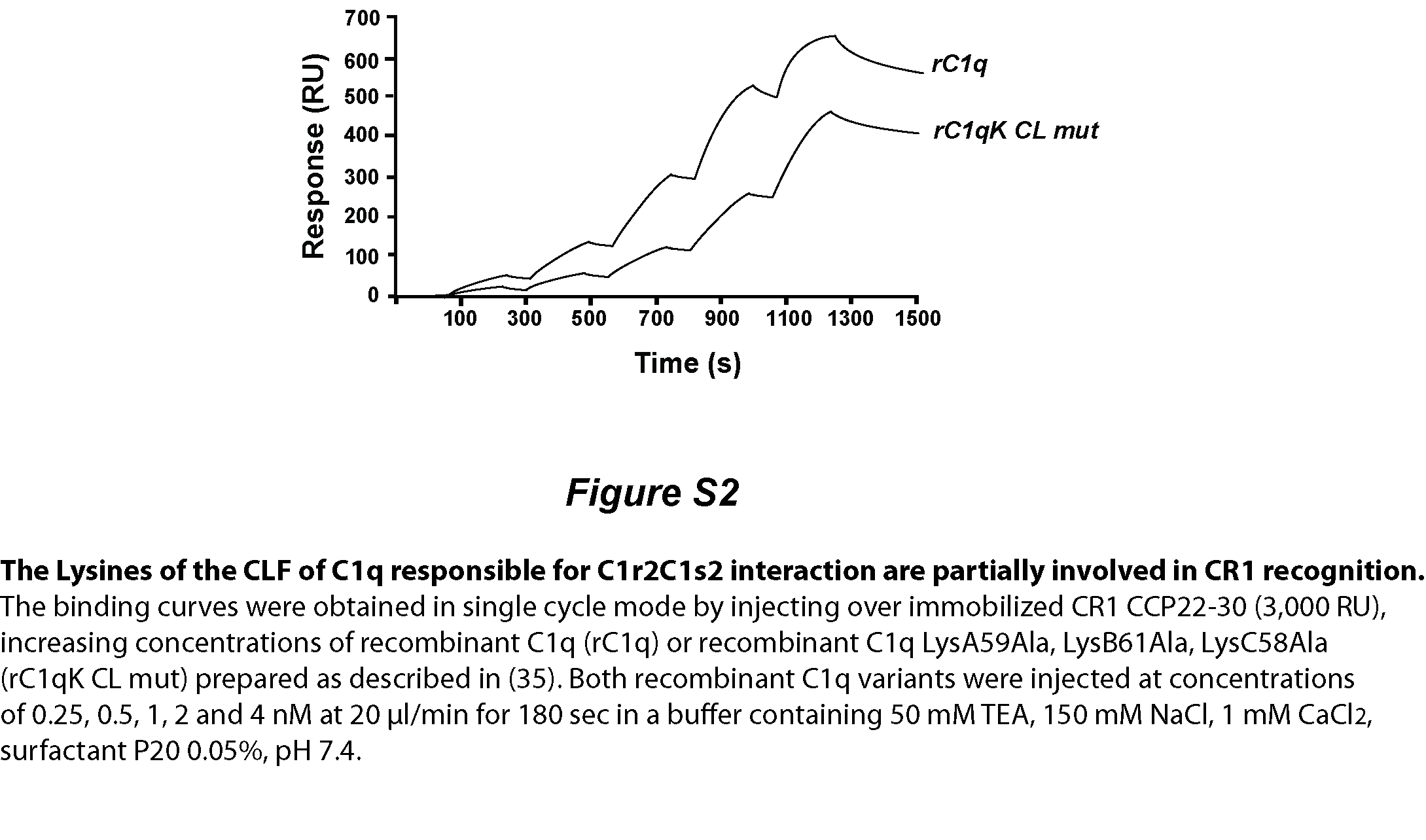

Supplement: Supplementary file 2 [file image_2.tif]
